# Effects of Distracting Task with Different Mental Workload on Steady-State Visual Evoked Potential Based Brain Computer Interfaces—an Offline Study

**DOI:** 10.3389/fnins.2018.00079

**Published:** 2018-02-15

**Authors:** Yawei Zhao, Jiabei Tang, Yong Cao, Xuejun Jiao, Minpeng Xu, Peng Zhou, Dong Ming, Hongzhi Qi

**Affiliations:** ^1^Department of Biomedical Engineering, College of Precision Instruments and Optoelectronics Engineering, Tianjin University, Tianjin, China; ^2^National Key Laboratory of Human Factors Engineering, China Astronaut Research and Training Center, Beijing, China

**Keywords:** brain–computer interface, SSVEP-BCI, mental workload, n-back, distracting task

## Abstract

Brain-computer interfaces (BCIs), independent of the brain's normal output pathways, are attracting an increasing amount of attention as devices that extract neural information. As a typical type of BCI system, the steady-state visual evoked potential (SSVEP)-based BCIs possess a high signal-to-noise ratio and information transfer rate. However, the current high speed SSVEP-BCIs were implemented with subjects concentrating on stimuli, and intentionally avoided additional tasks as distractors. This paper aimed to investigate how a distracting simultaneous task, a verbal n-back task with different mental workload, would affect the performance of SSVEP-BCI. The results from fifteen subjects revealed that the recognition accuracy of SSVEP-BCI was significantly impaired by the distracting task, especially under a high mental workload. The average classification accuracy across all subjects dropped by 8.67% at most from 1- to 4-back, and there was a significant negative correlation (maximum *r* = −0.48, *p* < 0.001) between accuracy and subjective mental workload evaluation of the distracting task. This study suggests a potential hindrance for the SSVEP-BCI daily use, and then improvements should be investigated in the future studies.

## Introduction

Brain-computer interfaces (BCIs), which allow individuals to communicate independently of the brain's normal output pathways of peripheral nerves and muscles, have attracted increasing amounts of attention in recent years. Individuals with motor disabilities can control external devices effectively using BCI systems that decode different patterns of electroencephalography (EEG). As a typical EEG pattern in BCIs, a steady-state visual evoked potential (SSVEP) is elicited by a visual stimulus blinking at a frequency higher than 6 Hz (Vialatte et al., 2010; Luis Fernando and Jaime, 2012). An SSVEP can be obviously recorded in the visual cortex (especially the V1 area) as a nearly sinusoidal oscillatory waveform with the same fundamental frequency as that of the stimulus, and often includes certain higher harmonics (Liu et al., 2014). When subjects use SSVEP-BCIs, multiple flickers with different stimulation properties (e.g., frequency and phase) were shown in the screen, and each of them encodes a different command (e.g., wheelchair movement). Subjects select one of the commands by focusing on one of the flickering stimuli, and related frequency or phase components would be modulated in their recorded EEG. By analyzing the generated SSVEP, the BCI system tries to identify which stimulus the subject selected.

Recently, SSVEP-BCIs have received increasing attention in BCI research due to their high signal-to-noise ratio (SNR) and information transfer rate (ITR). Furthermore, they can provide a large number of target sets and require little or even no extensive user training. Nakanishi and Wang et al. (Nakanishi et al., 2014) built a high-speed 32-target SSVEP-BCI by mixing frequency and phase coding with a computer monitor. Canonical correlation analysis (CCA) and SSVEP training data were combined for target detection, which permitted the average online ITR to reach 166.91 bits/min. Chen et al. (2015a) designed a filter bank canonical correlation analysis (FBCCA) to detect SSVEP without training data. In addition, an online ITR of 151.18 ± 20.34 bits/min was obtained under a 40-target frequency coding SSVEP-BCI. Subsequently, Chen et al. (2015b) designed a 40-character speller and developed a user-specific target identification algorithm using individual calibration data, reaching 60 characters (~12 words) per minute. Furthermore, SSVEP-BCIs have been used for clinical applications and could also be suitable for patients suffering from amyotrophic lateral sclerosis (ALS) (Hsu et al., 2016), locked-in syndrome (Combaz et al., 2013; Lesenfants et al., 2014), and other forms of paralysis (Muller et al., 2015; Lin and Hsieh, 2016). Lin and Hsieh (2016) implemented a low-cost wireless SSVEP-BCI for paralyzed patients to control several devices in a living room, and obtained an acceptable performance. Combaz et al. (2013) compared both P300- and SSVEP-BCIs for patients with locked-in syndrome, and found the SSVEP-BCI to be a faster, more accurate, less mentally demanding, and more satisfying BCI.

Nevertheless, the existing SSVEP-BCIs, especially high-speed BCIs, provide performances that are almost satisfactory under solely laboratory situations. Under such conditions, other brain activities due to distracting mental tasks were avoided, and only the EEGs evoked by the experimental procedures were recorded and analyzed. However, when using a BCI to output information, the human brain thinks and generates information, then the brain controls the BCI for output. These mental activities occur simultaneously. This process is similar to the thinking and writing process, in which the human brain runs two processes. Both of these processes compete with each other for its resources, and different levels of mental tasks will lead to varying degrees of interference. In the example of the thinking and writing process, the writing efficiency is different when the focus is on an easy word compared to a difficult one, and the possibility of writing errors also varies. Recently, the researchers studied the BCI efficiency in which the subjects performed both the BCI task and an additional mental activity. Researchers Kaethner et al. (2014); Ke et al. (2016) have found that the variation of mental states significantly affected the performance of event-related potential (ERP)-based BCIs. We previously published a paper (Ke et al., 2016) covering an investigation into the effect of mental activities on ERP-BCIs by verbal n-back stimuli during the training and testing of ERP-BCIs. The results showed that the temporal-occipital N200, the late reorienting negativity component, and features for classification decreased with an increase in mental workload. As expected, the performance of the ERP-BCI declined with rising levels of mental workload of the distracting task. Moreover, when classifiers were built under conditions of high mental workload, the performance was significantly improved as opposed to the previous performance under the speller-only conditions. Another study on two BCI tasks (Frenzel et al., 2011) designed a matrix speller where eye fixation and attention were separated for the purposes of ERP detection. The results showed that the performances of either task can be realized independently, however the performance of the BCI task would be disturbed when the distracting task was performed.

As the performances of SSVEP-BCIs are acceptable for application in some environments, we address them in this paper using a paradigm that adds another distracting task to an SSVEP-BCI. In terms of signal types, as for BCI control, the most important component in an SSVEP should be the stimulus-evoked potential, which is an exogenous signal that is determined by external physical stimuli and less susceptible to perceptual and cognitive processes. Therefore, an SSVEP-BCI would not be affected easily by a distracting task. However, considering the attention competition and feedback procedures in SSVEP-BCI, the SSVEP in this condition should contain the results after cognitive processing. Although the relationship between SSVEP and ERP is unclear, certain researchers (Capilla et al., 2011) suggested that visual steady-state responses can be explained as a superposition of transient ERPs. Moreover, correlations between certain ERP components, such as the N100 (Kramer et al., 1995; Ullsperger et al., 2001; Allison and Polich, 2008), the N200 (Kramer et al., 1995), and the positive–negative component around 200 ms (Missonnier et al., 2007; Pratt et al., 2011), and cognitive loads have been found. As a whole, however, there is insufficient evidence of the presence of weakened SSVEP-BCI effects suffered by distracting task, and this is what we propose to investigate in this paper. According to these backgrounds, we hypothesize that an SSVEP-BCI may be affected by a distracting task.

## Methods

### Participants and tasks

Fifteen healthy subjects (seven males, mean age: 23.5 ± 1.0 years) voluntarily participated in the experiment and provided informed consent before participating. The study was approved by the institutional review board of Tianjin University and the ethics committee of school of precision instrument and opto-electronics engineering. All subjects were right-handed, with normal or corrected-to-normal vision. None of them had a history of psychiatric or neurological disorders.

The subjects were seated ~70 cm from a computer monitor in a room illuminated with sufficient daylight. The stimulus program was developed by Psychophysics Toolbox Version 3 (Brainard, 1997) under MATLAB and presented on a 21.5-inch LCD screen with a resolution of 1,920 × 1,080 pixels and a refresh rate of 60 Hz.

In this paper, a parallel task experiment was constructed using an SSVEP-BCI and verbal n-back working memory task. Subjects performed a reactive SSVEP-BCI task where they used their attention to gaze the target stimuli by guided. The distracting n-back task played a role that interfered with subjects operating the SSVEP-BCI, and was unrelated to the BCI task. As one of the most popular experimental paradigms for studies on working memory, n-back tasks require subjects to monitor the identity of a series of verbal stimuli (e.g., letters and words) or nonverbal stimuli (e.g., shapes, faces and pictures) and indicate when the currently presented stimulus is the same as the one presented *n* trials earlier (Owen et al., 2005). For n-back tasks, *n* is a controlled variable to make subjects experience different levels of mental workload. In the whole period of an n-back task, subjects should keep remembering number *n*, and a sequence update is needed when new *n* occurs. In our experiment, verbal 1-, 3-, and 4-back tasks were performed, which presented three rising levels of mental workload conditions and depicted the intensity of the interference.

**Figure 1:**
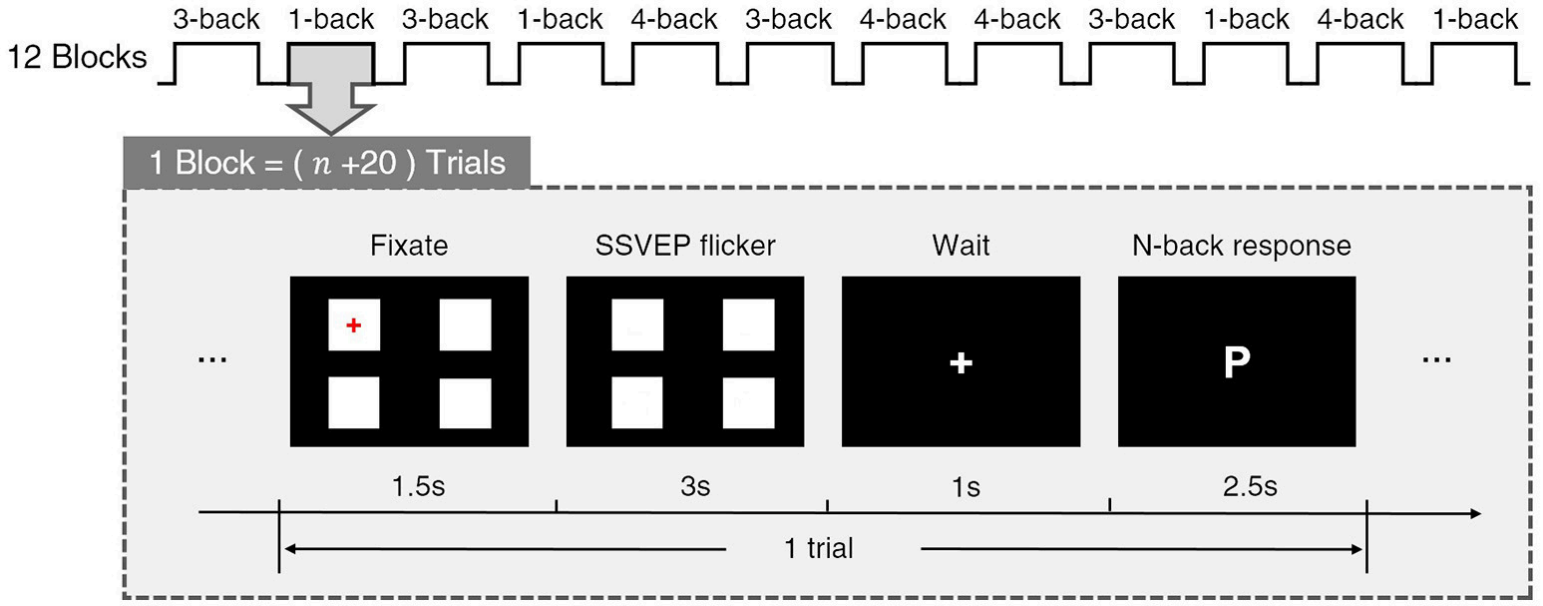
Experimental procedure. During SSVEP flicker period, four stimulus squares flickered with the frequency of 7, 11, 13, and 15 Hz respectively.

As shown in Figure 1, each condition comprised four blocks and all twelve blocks were presented in a random sequence. Each block was composed of (*n* + 20) trials (*n* = 1,3,4), and the last 20 trials were used for responses and further analysis in n-back tasks. At the beginning of each trial, four white squares of 100 × 100 pixels were placed at a distance of 200 pixels from each other vertically and horizontally on a black background. Participants were required to fix their eyes on the target square, which had been marked with a red cross (1.5 s). Four stimulus squares then flickered at 7, 11, 13, and 15 Hz, and SSVEPs were provoked by the target square flickering (3 s). After the flicker finished, a cross was presented at the center, and subjects were required to turn the gaze to it and wait for 1 s. Then a letter was presented at the center, and subjects were asked to compare the letter with the former *n*th (*n* = 1,3,4) letter and decide whether they matched by pressing the “left” (positive) or “right” (negative) keys as quickly as they could (2.5 s). Regarding the 3-back block, for example, if the letters presented in the prior three trials were “W,” “I,” and “S,” and the fourth trial showed “W,” the “left” key would have to be pressed to indicate the fact that there was a match with the first trial, and if the fifth trial showed “K,” the “right” key would have to be pressed to indicate the fact that there was no match with the second trial. In each block, 50% of the trials matched while 50% did not match. The response accuracies and response times were recorded and further analyzed for behavioral analysis. At the end of each block, subjects were asked to complete a rating scale mental effort (RSME) (Verwey and Veltman, 1996) to evaluate their mental workload with a score from 0 to 150 in the previous block. Subjects were then prompted to take a break for at least 1 min. In a total of 12 blocks, 20 trials of SSVEP data were obtained for each of 4 frequency stimuli and each n-back condition. It is important to emphasize that when the SSVEP-BCI task is executed, the characters in the n-back task are not displayed, which avoids the problem of the prompted gaze shift.

In brief, subjects were ensured to complete two tasks at the same time in this experiment. The first task is the usual SSVEP-BCI task, that is, subjects should use their attention to select the target stimulus frequency. The second task is a distracting task, the n-back task, and the bigger the n, the more attention it needs to be devoted in this task. Then, in this experiment, the subject could not put all his attention into the BCI task and his attention would be affected by the distracting task. The performance of the SSVEP-BCI operation is evaluated by the classification accuracy, and the situation of different mental workload of distracting tasks is compared.

### EEG data acquisition and preprocessing

Subjects were fitted with a Neuroscan 40-channel Quik-Cap, using 32 Ag/AgCl scalp electrodes, positioned according to the international 10/20 system. Electrode impedances were kept below 5 kΩ. EEG data were acquired using a Neuroscan NuAmps system at a sampling frequency of 1,000 Hz. Electrodes were initially referenced to the right mastoid (A2 channel) and grounded to the vertex.

During the preprocessing of the raw data obtained from the EEGs, data were re-referenced to the Fz channel at first. In this way, more obvious SSVEPs would be observed in the visual cortex, and higher classification accuracies would be achieved. A third-order band-pass filter from 6 to 80 Hz was then used for the data from each channel. The filter was a zero-phase Chebyshev type I infinite impulse response (IIR) filter, and it was implemented by the filtfilt() function in MATLAB. For each block, EEG data epochs comprising SSVEP signals (3 s in total) were extracted according to event labels generated by the stimuli. According to previous research (Di Russo and Spinelli, 1999), a latency delay due to the visual system should be considered, and the SSVEP data was extracted from 0.12 to 3.12 s after the SSVEP stimuli began. For an improved discrimination accuracy, the O1, Oz, and O2 channels were selected for further SSVEP detection. To summarize, for each of the three n-back conditions, 80 trials of SSVEP signals of 3 s were used for further identification, which maintained an equal trial number for each frequency.

### SSVEP amplitudes and recognition

#### Amplitude and signal-to-noise ratio of SSVEPs

For better presentation of the SSVEP harmonic components, the amplitude spectra and SNRs were calculated as features in the frequency domain for SSVEP. In this study, the SSVEP raw data were initially processed to remove the baseline drift. Then, for every trial, an SSVEP of 3 s was used for calculating the amplitude spectrum *y*(*f*) using a 3,000-point fast Fourier transform (FFT). The SNR was defined as the ratio of *y*(*f*) to the mean value of the neighboring frequency bands:

$$\begin{array}{l} \text{SNR}=20\text{lo}{\text{g}}_{10}\frac{K\times y\left(f\right)}{\sum_{k=1}^{K/2}\left(y\left(f+k\Delta f\right)+y\left(f-k\Delta f\right)\right)} \end{array}$$

Where Δ*f* is the frequency resolution in the amplitude spectrum. In this study, Δ*f* is 0.33 Hz and K is set to 6. For each trial, the amplitude spectrum of SSVEP was estimated and then used to calculate the SNR. The SNR was then averaged across 20 trials for each frequency.

#### SSVEP recognition methods

In this paper, data containing SSVEP epochs after preprocessing were used for detection to evaluate the BCI performance under distracting task with diverse mental workload. As for the identification of SSVEP, CCA was first used for multichannel SSVEP detection by Lin et al. (2007) and has been regarded as a typical algorithm. Several similar methods based on CCA (Bin et al., 2009; Chen et al., 2014, 2015a,b; Nakanishi et al., 2014) have been published for SSVEP detection and extraordinary ITRs have been achieved. Considering that the BCI performance might also be influenced by the classification method, this might lead to limited results when using just one single method. As a comparison, three kinds of recognition methods were used for the classification of SSVEP, and the common trend was expected to be found:

- *Standard CCA*. CCA is a multivariable statistical method used when there are two sets of data between which exists a degree of underlying correlation (Lin et al., 2007). Canonical correlations between multichannel SSVEPs and reference signals on each stimulus frequency were calculated in this paper. The frequency of reference signals possessing the maximum correlation is regarded as the frequency of the target SSVEPs.
- *Filter bank CCA (FBCCA)*. Filter bank analysis is used to decompose SSVEPs into sub-band components, such that independent information embedded in the harmonic components can be extracted more efficiently for enhancing the detection of SSVEPs (Chen et al., 2015a). The filter bank method consists of three major procedures: (i) sub-band decomposition, (ii) feature extraction for each sub-band signal, and (iii) target identification by CCA.
- *SSVEP template-based CCA (TCCA)*. Individual calibration data has been used as individual SSVEP templates in reference signals to improve target detection (Nakanishi et al., 2014). In the paper by Nakanishi, the training SSVEP template signal that maximized the weighted correlation value was selected as the SSVEP template signal corresponding to the target. In our analysis, classifiers of different n-back tasks were all trained on 1-back data and were then used to test 1-, 3-, and 4-back data.

### Statistical analysis

In this paper, we used SPSS software (IBM SPSS Statistics, IBM Corporation) to perform statistical analysis. A One-way analysis of variance (ANOVA) was used to test the difference of behavior and classification accuracies among different n-back conditions. The welch correction was applied if the data did not conform to the normality assumption by the homogeneity of variance test. The *post-hoc* pairwise comparisons were Bonferroni corrected. A paired *t*-test was applied to compare the difference between the 1- and 4-back in SNRs. The alpha was set to 0.05.

## Results

### Behavior and RSME

**Figure 2:**
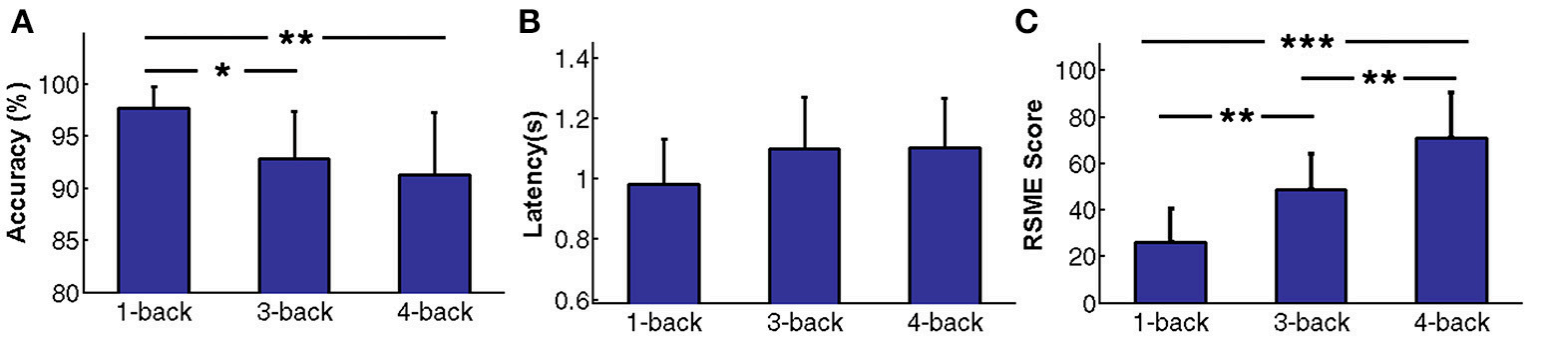
Behavior performance and RSME scores for n-back tasks**. (A)** Response accuracy. **(B)** Response latency. **(C)** RSME scores. The error bars represent standard deviations across subjects and asterisks represent significant differences between two n-back tasks (^*^p < 0.05, ^**^p < 0.01, ^***^p < 0.001).

The behavior performance of the n-back tasks is shown in Figures 2A,B, where the error bars represent the standard deviations across subjects. The response accuracies were 97.75±2.07%, 92.83 ± 4.57%, and 91.33 ± 6.02% for the 1-, 3-, and 4-back tasks, respectively. The reaction latencies were 0.98 ± 0.15 s, 1.10 ± 0.17 s, and 1.10 ± 0.16 s for the 1-, 3-, and 4-back tasks, respectively. By one-way ANOVA, although the difference in reaction time was insignificant [*F*_(2, 42)_ = 2.74, *p* > 0.05], a significant difference in accuracy was found between the n-back tasks [*F*_(2, 42)_ = 8.26, *p* < 0.001]. Furthermore, *post-hoc* pairwise comparisons showed that the response accuracy of the 1-back task was significantly higher than those of the 3-back (*p* < 0.05) or 4-back (*p* < 0.05) tasks. The same trend in RSME scores is shown in Figure 2C. The RSME scores were 26.30 ± 14.22, 48.78 ± 15.17, and 71.15 ± 19.55 for the 1-, 3-, and 4-back tasks, respectively. A one-way ANOVA revealed a significant difference between the RSME scores of the n-back tasks [*F*_(2, 42)_ = 27.78, *p* < 0.001]. Moreover, significant differences were found between the 1- and 3-back tasks (*p* < 0.01), the 1- and 4-back tasks (*p* < 0.001), and the 3- and 4-back tasks (*p* < 0.01) by *post-hoc* pairwise comparisons. The behavior and RSME results indicated that the subjects were under different mental load states in the different n-back experimental conditions.

### BCI performance under n-Back tasks

The classification accuracies under different n-back tasks were calculated by CCA, FBCCA, and TCCA. In TCCA, the classifiers were all trained on the 1-back data and then used to test the 1-, 3-, and 4-back data. For 1-back conditions, cross validation (leave-one-out) was used to partition the data into complementary subsets: training and testing sets. Twenty-fold cross-validation was performed, and validation results were averaged to calculate the final recognition accuracy.

**Figure 3:**
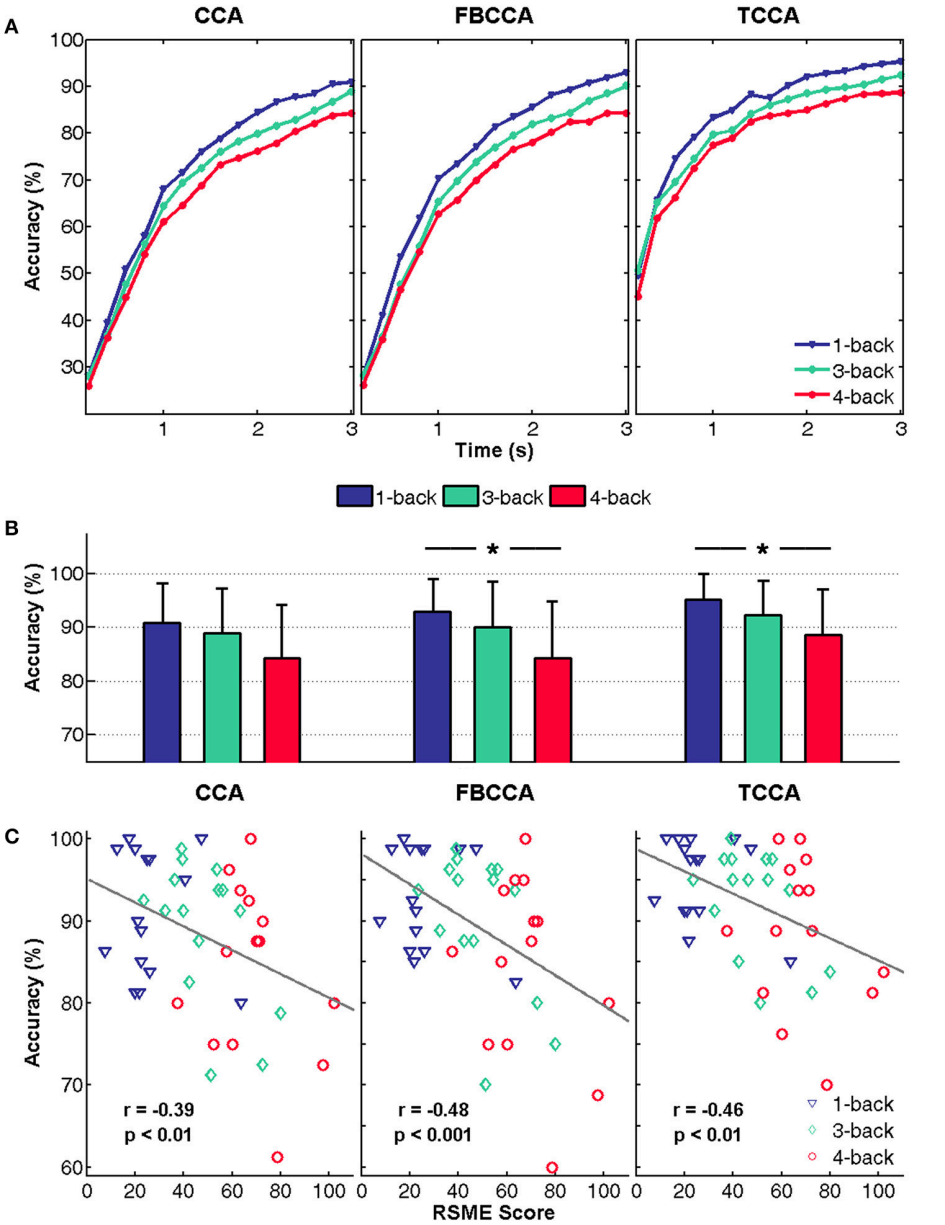
BCI classification accuracy. **(A)** Mean classification accuracies with various data lengths (from 0.2 to 3 s in steps of 0.2 s) for the 1-, 3-, and 4-back tasks. **(B)** Mean classification accuracy with a data length of 3 s. The error bars represent standard deviations across subjects and asterisks represent significant differences between two n-back tasks (^*^p < 0.05). **(C)** Correlation analysis between RSME score and accuracy with a data length of 3 s.

Figure 3A shows the SSVEP-BCI mean classification accuracies corresponding to different data lengths (from 0.2 to 3 s in steps of 0.2 s). In general, the classification accuracies were improved as the data length increased. It is clear that of the three methods, the accuracy of the 1-back task was the highest, followed by the 3-back task, with the 4-back task having the lowest accuracy.

Figure 3B shows a comparison of the classification accuracies at 3 s under the 1-, 3-, and 4-back tasks, and the error bars depict the standard deviation across subjects. As in Figure 3A, the SSVEP classification accuracy decreased significantly with the increase in n-back level. For FBCCA and TCCA, significant differences were found by one-way ANOVA [FBCCA: *F*_(2, 42)_ = 3.737, *p* < 0.05; TCCA: *F*_(2, 42)_ = 3.372, *p* < 0.05]. The *post-hoc* pairwise comparisons revealed a significant difference in classification accuracy at binary n-back levels (FBCCA: 1- vs. 4-back, *p* < 0.05; TCCA: 1- vs. 4-back, *p* < 0.05). These results imply that variations in the mental states of the users during distracting tasks significantly affected the performance of SSVEP-based BCI. Although no significant difference was found in CCA accuracies by one-way ANOVA, the paired *t*-test also showed significant difference in 1- and 4-back (*p* < 0.01). It should be noted that, for all methods, the difference between the 1- and 4-back tasks was larger and more significant than that between the 1- and 3-back tasks. On the one hand, this shows that the SSVEP-BCI performance remained relatively steady while an easy distracting task was performed. On the other hand, this finding suggests that performances will be significantly influenced when users experience a rather high level of mental workload of the distracting task.

Since variations in the effects of mental workload received from the same level of n-back tasks may exist among individuals, the RSME score was required to record the subjective experience under these tasks and to evaluate individual levels of perceived mental workload. The correlation between the RSME score and accuracy with a data length of 3 s was calculated using the Pearson correlation coefficient and is shown in Figure 3C. Figure 3C illustrates that the accuracies by all three methods were significantly negatively correlated with RSME scores (CCA: *r* = −0.39, *p* < 0.01; FBCCA: *r* = −0.48, *p* < 0.001; TCCA: *r* = −0.46, *p* < 0.01). This indicated that an increase in the level of mental effort experienced resulted in a more noticeable decline in identification results for individuals using the SSVEP-BCI while performing other tasks as distractors. The results further suggested that distracting tasks requiring a high level of mental effort would significantly impair SSVEP-BCI performance.

### Individual difference and grouping

Table 1 lists the maximum accuracy under the 1-, 3-, and 4-back conditions and the difference between the 1- and 4-back tasks for each subject. The maximum accuracy was the maximum SSVEP classification accuracy for the three methods with a data length of 3 s, and the arrow at the end of each row of the table indicates the trend in accuracy variation. As shown in Table 1, although the accuracy of most subjects (13/15) declined from the 1- to 4-back tasks, a clear difference among individuals in terms of the deterioration in accuracy was still noted. The difference in accuracy for six subjects exceeded 10%, while that for four other subjects was less than 3%. We divided the subjects into two groups according to the maximum difference in accuracy. We selected a total of seven subjects, S1, S5, S6, S8, S13, S14, and S15 (in gray in Table 1), for whom the differences in accuracy exceeded 5%, as the sensitive group in terms of distracting task (group S). The differences in accuracy for S5, S14, and S15 in group S actually exceeded 15%. The other eight subjects (S2, S3, S4, S7, S9, S10, S11, and S12), whose accuracy differences did not exceed 5%, were chosen as the insensitive group in terms of distracting task (group IS). Under the 1-back task, the average accuracy of group S (95.00 ± 5.64%) was approximately the same as that of group IS (96.09 ± 4.93%). The difference between the two groups gradually became larger and more significant with increasing n-back level. The one-way ANOVA analysis showed that, with increasing n-back level, the accuracy of group S [CCA: *F*_(2, 18)_ = 3.91, *p* < 0.05; FBCCA: *F*_(2, 18)_ = 5.73, *p* < 0.05; TCCA: *F*_(2, 18)_ = 4.76, *p* < 0.05] decreased significantly, whereas that of group IS [CCA: *F*_(2, 21)_ = 0.48, insignificant; FBCCA: *F*_(2, 21)_ = 0.42, insignificant; TCCA: *F*_(2, 21)_ = 0.41, insignificant] remained almost constant. The results showed that certain users were not affected by the distracting task when using the SSVEP-BCI. In contrast, for nearly half of the number of users (group S), the BCI performance deteriorated under conditions of high mental workload of the distracting task. As such, the following results were analyzed separately for the two groups.

**Table 1:** Classification accuracy.

**Subject**	**Maximum accuracy (%)**			**Difference between 1- and 4-back (%)**	
	**1-back**	**3-back**	**4-back**		
S1	98.75	97.50	88.75	10.00	↓
S2	91.25	87.50	90.00	1.25	↓
S3	87.50	83.75	83.75	3.75	↓
S4	100.00	95.00	97.50	2.50	↓
S5	85.00	81.25	70.00	15.00	↓
S6	100.00	93.75	90.00	10.00	↓
S7	100.00	97.50	95.00	5.00	↓
S8	92.50	80.00	81.25	11.25	↓
S9	98.75	97.50	100.00	−1.25	↑
S10	98.75	95.00	96.25	2.50	↓
S11	100.00	100.00	100.00	0.00	–
S12	92.50	95.00	88.75	3.75	↓
S13	100.00	97.50	93.75	6.25	↓
S14	91.25	95.00	76.25	15.00	↓
S15	97.50	91.25	81.25	16.25	↓
Mean ± S.D.	96.09 ± 4.93	93.91 ± 5.49	93.91 ± 5.84	2.19 ± 2.09	
Mean ± S.D.	95.00 ± 5.64	90.89 ± 7.35	83.04 ± 8.35	11.96 ± 3.60	

### The BCI performance on frequency

**Figure 4:**
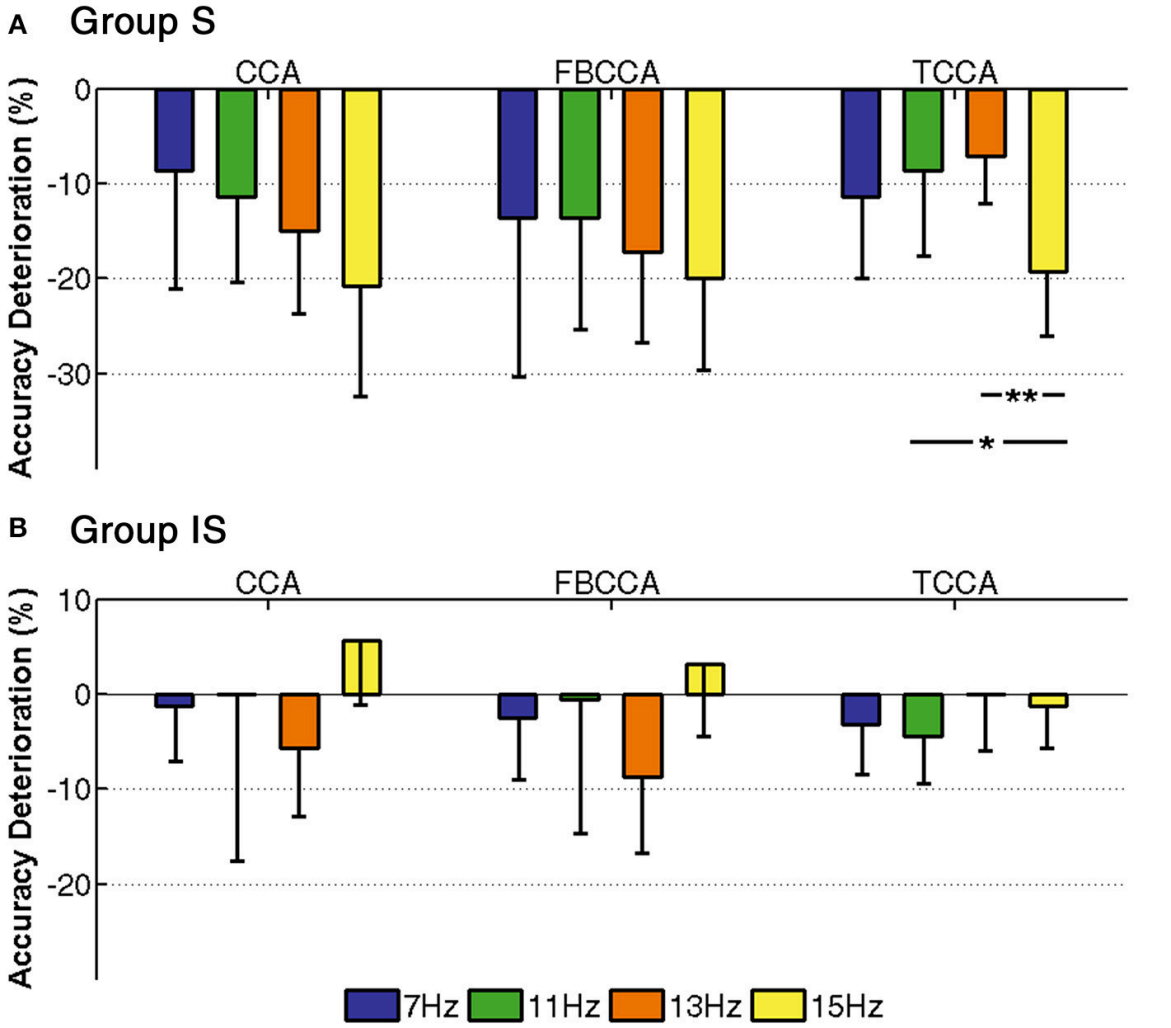
Deterioration of classification accuracy from 1- to 4-back with a data length of 3 s for each frequency. The error bars represent standard deviations across subjects and asterisks represent significant differences between two frequencies (^*^p < 0.05, ^**^p < 0.01). **(A)** Group S. **(B)** Group IS.

In order to further analyze the effect of various mental states on the SSVEP-BCI frequencies, the accuracy deterioration from the 1- to 4-back tasks is shown in Figure 4, where the error bars show the standard deviations across subjects. Upon comparing group S (Figure 4A) with group IS (Figure 4B), it is clear that the accuracy deterioration in group S was more severe than that in group IS at each frequency. As shown in Figure 4A, a one-way ANOVA showed that the accuracy deterioration was significant for TCCA [*F*_(3, 24)_ = 3.70, *p* < 0.05], whereas no significant difference was found for CCA or FBCCA. The difference between frequencies was only 2 Hz, and this was not significant enough for comparison by one-way ANOVA. In terms of frequency classification, 15 Hz belongs to the beta range (14–30 Hz), while both 11 and 13 Hz belong to the alpha range (8–13 Hz). In group S, certain significant increases in accuracy deterioration were found between 11 and 15 Hz (TCCA: *p* < 0.05), and 13 and 15 Hz (TCCA: *p* < 0.01). In contrast, for group IS in Figure 4B, a one-way ANOVA showed the deterioration of the accuracy was insignificant by all three methods [CCA: *F*_(3, 28)_ = 1.56, insignificant; FBCCA: *F*_(3, 28)_ = 2.20, insignificant; TCCA: *F*_(3, 28)_ = 1.12, insignificant]. The results for group S suggested that by each method, the deterioration of accuracy from the 1- to 4-back tasks was more severe with increasing frequency. It may be observed that SSVEP at higher frequencies is more sensitive to the mental workload of the distracting task, and SSVEP at lower frequencies is more stable and therefore recommended for BCI systems.

### SNR of SSVEPs

**Figure 5:**
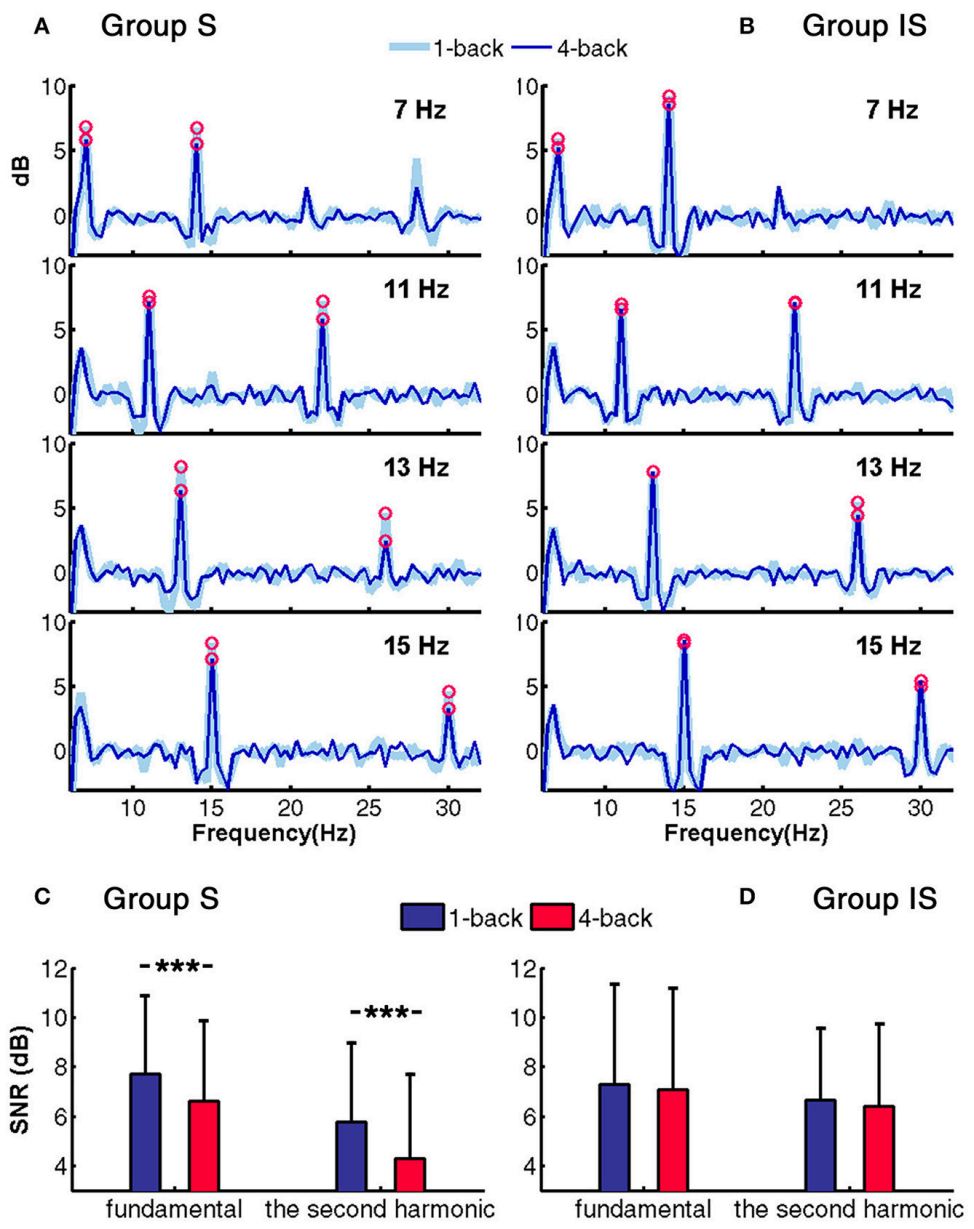
SNR of SSVEPs on the Oz channel for the 1- and 4-back tasks. The red circles indicate the SNRs of the fundamental and second harmonic frequencies for each target SSVEP. **(A)** Group S. **(B)** Group IS. Mean SNR of all fundamental and second harmonics. The error bars represent the standard deviations across subjects and frequencies and asterisks represent significant differences between two n-back tasks (^***^p < 0.001). **(C)** Group S. **(D)** Group IS.

To examine the differences in SSVEP with the change in mental workload of the distracting task, for each stimulus frequency, the SNRs of the SSVEPs were estimated by averaging across the group. Figures 5A,B show the comparison of the mean SNR of SSVEPs at the first and second harmonics between the 1- and 4-back tasks in groups S and IS. For the two groups, both the first and second harmonics could be clearly observed in the SNR. In group S, SNR of each target harmonics dropped obviously from 1- to 4-back tasks. However, in group IS, most of the SNR of harmonics remained relatively stable during the 1- and 4-back tasks, except the first harmonic at 7 Hz and the second harmonic at 7 and 13 Hz. Figures 5C,D compare the mean SNR between the 1- and 4-back tasks at the first and second harmonics for groups S and IS, respectively. The SNRs were averaged by subjects and four frequencies, and the error bars represent the standard deviations across subjects and frequencies. In group S, the paired *t*-test showed a significant difference for the 1- and 4-back tasks in both fundamental (*t* = 3.73, *p* < 0.001) and the second harmonic (*t* = 4.12, *p* < 0.001). Meanwhile, no significant difference was found for group IS. The result from SNRs suggested the reduction in SSVEP-BCI accuracy and SSVEP feature was consistent.

However, the classification accuracy and SSVEP feature in two groups were not completely consistent, this might be caused by the difference between two groups in BCI performance. And the limitation of the subject number and inter-subject difference also caused the statistical result not that significant.

## Discussion

In this paper, the performances of SSVEP-BCIs were found to deteriorate significantly by distracting task, especially under the 4-back conditions. Moreover, for certain subjects such as those in group S, the SNR of SSVEP harmonics dropped significantly under conditions of high mental workload. However, the SSVEP features presented a less depressing trend compared to the performance of BCI. We speculate that a BCI system comprises neural feedback, attention competition and other responses, and the performance of it would be affected by multiple factors. While the EEG features were only in one channel and one harmonic, unable to present the comprehensive information significantly.

In a previous study (Ke et al., 2016), we discussed the effect on P300-Speller when users were under mental workload conditions. To continue the study into the effect of distracting task on BCIs, we proposed studying the performance of other BCI systems with distracting task. It should be pointed out that the reduction in accuracy was not as significant as for P300-Speller under 3-back conditions. This observation is reflected in the following two points:

- *Effects on accuracy*. Previous results (Ke et al., 2016) have shown that the decrease in accuracy from the speller under only 3-back conditions was almost 10% and remained consistent with an increasing number of repetitions. However, in this paper, when subjects experienced the 3-back task under conditions of high levels of mental load, the reduction in accuracy was not significant for each method, and the accuracy decrease of 3% did not cause severe impairment of BCI performance. From this point of view, compared to P300-BCI, the SSVEP-BCI performed better when subjects were under low levels of mental workload of the distracting task.
- *Individual differences*. The previous results (Ke et al., 2016) showed that almost all subjects were affected by the distracting task. The classification accuracy displayed a significant decreasing trend and there was relatively little individual difference. However, a significant individual difference was found for the SSVEP-BCI performance.

The apparent higher stability of the SSVEP-BCI with distracting task warrants further discussion. Firstly, the P300 component as a typical component in an ERP is an endogenous component, which can be easily influenced by distracting perceptual and cognitive tasks. However, as an exogenous component, SSVEP should be less susceptible to individual perceptual and cognitive processes. This characteristic also increases the robustness of SSVEP-BCIs and maintains a high SNR. Secondly, the classifier in P300 identification must be calibrated using individual data. The difference in the distribution of n-back EEG features means that the classifier has a different generalization performance, causing a significant decline in classification. Both CCA and FBCCA are subject-independent algorithms for SSVEP classification and classifier calibration is not required. This means that the classification process is not easily affected by the diverse feature distributions. Furthermore, the three methods for SSVEP detection are all based on frequency correlation analysis, which is insensitive to changes in EEG power. This may also result in an SSVEP-BCI not being as sensitive to mental load states. However, the distracting task we discussed in this paper had no correlation with the BCIs. For clinical use, other daily workload tasks like thinking and gazing have more complex correlations with SSVEP-BCIs, which may lead a severe impairment in the performance of SSVEP-BCIs.

In this paper, the performance of SSVEP-BCIs was found to be weakened by the distracting task, and this finding may have a relationship with the top-down modulation. Intaite et al. (2014) found that general top-down modulation had an influence on visual processing, possibly mediated by the prefrontal cortex. This meant that brain responses in the occipital areas at 150–300 ms post-stimulus were influenced by working memory load. Furthermore, a previous study (Gazzaley, 2011) revealed that the top-down modulation of visual cortical activity at early perceptual processing stages influences the subsequent working memory performance. As a stable and high SNR response signal, SSVEP is always used as a steady-state “topographical probe” (SSTP), a frequency tag associated with a visual task (Silberstein et al., 1990, 1995). Several studies (Silberstein et al., 2001; Ellis et al., 2006; Cao et al., 2014) have used SSVEP frequency propagation during task vs. control states to indirectly estimate the propagation of EEG signals related with the task (Vialatte et al., 2010). Ellis et al. (2006) examined changes in temporal neurophysiology during spatial n-back tasks using SSPT. They found that the delay period (or information-holding period) was associated with increases in frontal and occipital region amplitude, consistent with previous findings in more basic working memory tasks, and no correlation between SSVEP and performance was observed. However, similar results were not obtained in this paper, because of the difference in paradigm design and process method. On the one hand, BCI recognition summed up EEG features from several channels and multiple harmonics, while we only analyzed the EEG features in one channel and one harmonic. It is therefore not comprehensive to value SSVEP-BCI performance simply by the SSVEP feature in a single channel. On the other hand, there was a correlation of the competition between the multi-frequency SSVEPs when the SSVEP-BCI was used. Studies into the working memory using SSPT only presented SSVEP stimuli with one frequency to the subjects, and no competition in frequency was involved during working memory research.

## Conclusion

This study examined the effects of distracting task on SSVEP-BCI performance in an offline study and explored the influence on the characteristic components of SSVEP. The results indicated that simultaneous distracting task significantly impaired SSVEP-BCI performance. Moreover, the subjects whose accuracy was clearly influenced by distracting task also exhibited a clear decline in the SNR of SSVEP. This study suggests that BCIs as information pathways of the human brain are restricted by workload mental processes, which should be improved upon for future applications.

## Author contributions

YZ: Performed the experiment and wrote manuscript; JT: Offered the analysis methods; YC and XJ: Helped to improve the paper; MX: Helped to improve the method; PZ and DM: Contributed to the experiment and discussion; HQ: Conceived and designed the study.

### Conflict of interest statement

The authors declare that the research was conducted in the absence of any commercial or financial relationships that could be construed as a potential conflict of interest.
